# Innovative microfluidic model for investigating the intestinal mucus barrier: numerical and experimental perspectives

**DOI:** 10.1007/s13346-025-01818-8

**Published:** 2025-03-06

**Authors:** Mohammad Valibeknejad, Reza Alizadeh, S. Majid Abdoli, Julian Quodbach, Faranak Heidari, Silvia M. Mihăilă, Pouyan E. Boukany, Amir Raoof

**Affiliations:** 1 Department of Earth Sciences, Utrecht University, Utrecht, the Netherlands; 2 Department of Chemical Engineering, Sahand University of Technology, Sahand New Town, Tabriz, Iran; 3 Division of Pharmaceutics, Utrecht Institute for Pharmaceutical Sciences, Utrecht University, Utrecht, the Netherlands; 4 Division of Pharmacology, Utrecht Institute for Pharmaceutical Sciences, Utrecht University, Utrecht, The Netherlands; 5 Department of Chemical Engineering, Delft University of Technology, Delft, The Netherlands

**Keywords:** Intestinal barriers, Microfluidic, Biosimilar mucus (BSM), Rheology, Computational fluid dynamics (CFD)

## Abstract

The intestinal mucus layer serves as a critical first line of defense against external agents, functioning as a barrier to the absorption of drugs, food, and pathogens. While numerous in vitro studies have explored the role of mucus in preventing particle penetration, the effects of flowing luminal material, dislodging of mucus because of induced shear rate by lumen material and interfacial phenomena remain poorly understood. This study introduces a microfluidic approach to simulate the interaction between flowing luminal material and the mucus layer. The approach successfully measures both particle penetration into the mucus layer and the rate of mucus dislodgement by flowing luminal material. A biosimilar mucus model (BSM) and Hank's Balanced Salt Solution (HBSS) were employed as mimics of human intestinal mucus and luminal fluid, respectively. To investigate the effect of viscosity on the particle penetration pattern, two variants of the mucus model were used: BSM-1, representing a low-viscosity mucus model, and BSM-2, representing a high-viscosity mucus model. The velocity fields in the mucus and luminal material were extracted by tracking fluorescent particles. The results revealed significant differences between BSM-1 and BSM-2, attributed to their rheological properties. These findings were further confirmed through an assessment of the viscoelastic properties of the BSM models. The study utilized COMSOL Multiphysics for numerical simulations, successfully predicting experimental outcomes by solving fluid flow equations. Physicochemical characterizations of BSM and HBSS were performed to link the experimental results with numerical simulations, including flow sweep tests, the application of the power-law model for viscosity, and measurements of mucus density and wettability. This study proposes a microfluidic platform for examining mucus dislodgement and particle penetration in both low- and high-viscosity mucus models. The findings offer valuable insights into the intestinal mucus barrier's response to shear stress. The validated numerical approach and physicochemical characterizations provide a foundation for future studies on mucus dislodgement rates and penetration in more complex intestinal geometries and diverse flow conditions.

**Graphical Abstract:**

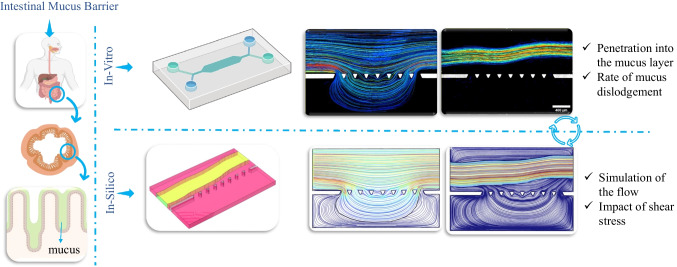

## Introduction

Mucous membranes, located in various organs, such as the gastrointestinal and respiratory tracts, and the vagina, are pivotal in defending against pathogens, primarily through mucus secretion. This mucus acts as a protective barrier against external threats, but also presents challenges for the absorption of drugs and nutrients in the intestine [1–7]. To increase absorption, techniques such as mucus clearing,‌ mucus weakening, and drug carrier modification [8–15] are investigated. In silico and in vitro assessments of these techniques and new drug discoveries are required before proceeding to in vivo testing. An in vitro platform for evaluating the barrier properties of the mucus layer allows for the simulation of physiological conditions, such as specific diseases, the presence of bacteria, and different segments of the intestine. Additionally, in silico approaches can predict drug absorption through intestinal mucus under complex geometries and flow conditions.

Traditional static chamber systems, widely used to study the mucus barrier [16–18], lack key dynamic aspects of the intestinal environment, such as residence time and shear stress from luminal flow [19, 20]. Recent innovations in microfluidic systems have led to more physiologically relevant models by incorporating these dynamic features. Notable studies have demonstrated the use of microfluidic chips to investigate transport processes, and drug carrier interactions [21–28]. However, many existing designs still lack crucial factors such as flow dynamics, direct exposure of lumen material to the mucus layer, mucus retention, and long-term stability, limiting their effectiveness in simulating the intestinal mucus barrier.

In addition to experimental approaches, numerical modelling has proven complementary by simulating fluid flow and mass transfer processes across the mucus layer [19]. Nevertheless, the accuracy of numerical simulations is highly dependent on the accurate parametrisation and validation against experimental data, which necessitates measuring the physicochemical properties of mucus and identifying appropriate modelling approaches for simulating flow within the mucus layer.

This study aims to overcome these limitations by developing a microfluidic platform that replicates physiologically relevant conditions. The primary objective is to simulate the dynamic interaction between intestinal mucus and luminal material, enabling real-time assessment of particle penetration and mucus dislodging. To achieve this, a microfluidic device was designed with consideration of the wettability of mucus and luminal material, as well as interfacial forces, to accurately simulate the interaction between luminal flow and the mucus barrier. The device utilizes biosimilar mucus models (BSMs) with tunable rheological properties to represent various physiological and pathological conditions of the intestine and proposes an appropriate method for measuring penetration in both low- and high-viscosity mucus models. In parallel, a computational modelling framework was employed to simulate fluid flow and particle transport, facilitating a quantitative interpretation of the experimental data. To bridge the gap between experimental observations and numerical simulations, a mathematical model for BSM viscosity was introduced. This integrated experimental and computational approach provides a comprehensive platform for advancing the study of the intestinal mucus barrier under controlled and reproducible conditions.

## Material and methods

**Fig. 1:**
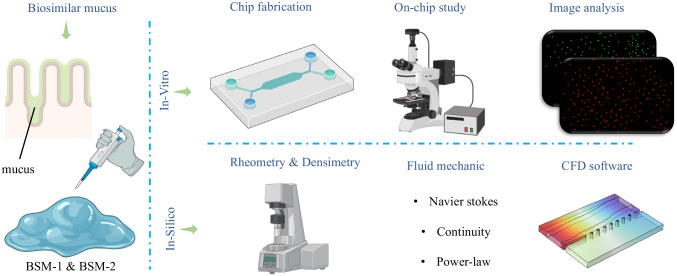
**Schematic overview of the study: Biosimilar mucus**: In this study, we employed synthetic mucus to replicate human intestinal mucus. Two series of synthetic mucus were utilized to validate their suitability, with one series possessing a higher elastic modulus. ***In-vitro***: The in-vitro section of this study involved fabricating a microfluidic chip using soft lithography. To achieve the optimal design and experimental setup, various designs were tested. Upon identifying the optimal design and experimentation method, synthetic biosimilar mucus was used to saturate the chip. Subsequently, injection of HBSS was initiated. Imaging of fluorescent particles in both the mucus and HBSS was conducted using a fluorescent microscope. Subsequently, the tracking of these particles was performed using ImageJ software to evaluate the velocity field, penetration depth, and the dislodgement rate of mucus by HBSS flow. ***In-silico*:** To conduct the numerical study, the viscosity and density of the mucus layer were initially measured. A power-law model was introduced to characterize the viscosity of the mucus. The fluid flow equations were solved and visualized using COMSOL software. The numerical approach was validated against experimental observations to ensure its accuracy

The overview of the method utilized in this study is schematically illustrated in Fig. 1. Briefly, soft lithography was used to create a PDMS (polydimethylsiloxane)-based microfluidic device for experimentation. Two variants of BSM, standing for different viscosities, were used to mimic intestinal mucus. Hank's Balanced Salt Solution (HBSS) was used as the luminal material. On-chip observations were captured and analysed to correlate these observations with the underlying physical phenomena. Following this, a power law model was introduced to describe the rheological properties of the mucus layer. Applying COMSOL software, the mathematical equation describing the flow was simulated to predict the on-chip observation. Subsequently, the simulation results were compared with experimental observations to confirm the modelling approach and support on-chip observation.

### Reagents

Mucin (Type III), hydroxyethyl piperazine ethane sulfonic acid (HEPES) sodium salt, cholesterol (> 99%), and linoleic acid (> 99%) were acquired from Sigma. Phosphatidylcholine, and bovine serum albumin (BSA) fraction V were obtained from Merck. Polyoxyethylene sorbitan monooleate (Tween 80) was purchased from TCI, carboxylate-modified polystyrene particles from Polysciences and polyacrylic acid (Carbomer 974P) from Fagron.

### 2.2 Microfluidic device and fabrication

**Fig. 2:**
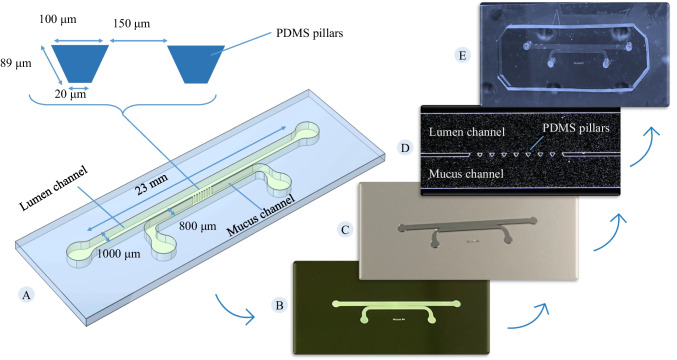
**Schematic representation of chip fabrication:** (**A**) CAD drawing illustrating the microfluidic design with two parallel channels and interfacing pillars. (**B**) Photomask created from the CAD design for photolithography. (**C**) Fabricated microfluidic design on a silicon wafer by photolithography technique. (**D**) Digital microscope image (VHX-5000, Keyence Corp) used for quality assessment of the mold. White lines indicate the borders of regions with cured photoresist, verifying the quality of the photolithography step with well-created edges. (**E**) Final microfluidic chip made of PDMS, bonded to glass slides, and ready for in-vitro analysis

To simulate the interaction between intestinal mucus and flowing lumen material, a microfluidic device was designed, as shown in Fig. 2-E. This design consists of two adjacent channels, one for lumen material and the other for mucus, separated by trapezoidal interfacing pillars. These pillars are aimed to provide strong capillary forces to retain the mucus in position and prevent leakage into the luminal channel (Fig. 2 panel A). To minimize end effects, a 10 mm segment was allocated for flow development and flow control pump parameter adjustment. The device design, shown in Fig. 2-A, was drafted using AutoCAD software (Version 2021 by Autodesk) and translated into a mask for photolithography (Fig. 2-B), with photomask production completed by JD Photo Data company. The silicon wafer substrate was cleaned with acetone and isopropanol for 5 min each, air-dried, and then baked for 30 min at 120°C to remove water. After cooling for 2 min at room temperature, the spin coater was set to reach 500 rpm with an acceleration of 100 rpm/sec for 7 s, and then 1700 rpm with an acceleration of 300 rpm/sec for 30 s. Su-8 2050 photoresist was applied to the wafer to achieve a 100 µm thickness. Following a soft bake at 65°C for 5 min and 95°C for 16 min, the wafer was cooled for 1 min at room temperature. The exposure required 230 mJ/cm^2^ to reach the desired thickness. Post-exposure, the wafer was baked at 65°C for 3 min and 95°C for 9 min, then developed by shaking in developer for 9 min and stopping the process with isopropanol. After washing and air drying, the wafer was heated on a hot plate at 150°C for 15 min to eliminate solvents (Fig. 2-C). Polydimethylsiloxane silicone elastomer and curing agent were mixed at a 10:1 ratio, degassed for 40 min, and poured onto the master mold. Curing was done in an oven at 60°C for 2 h. Once removed from the mold, inlet and outlet holes of 1.5 mm diameter was punched, and the device was assembled using microscopic slides, with the microscopic slides being bonded to the PDMS following treatment of both with corona discharge (Fig. 2-E).

### Preparation of biosimilar mucus (BSM) and lumen fluid mimic

Purified mucin-based hydrogels are commonly used in mucus studies due to their ease of preparation. However, they do not accurately replicate the rheological and barrier properties of native mucus [28, 29]. Biosimilar mucus models (BSM), based on proteomic and lipidomic analysis of native porcine intestinal mucus [30], have been developed to address this issue [31]. In this study, BSM was employed to replicate human intestinal mucus due to its accessibility and reproducibility. BSM was prepared as depicted in Table 1 based on the optimized methodology described by Boegh et al., with minor modifications [31]. The mucin solution was crosslinked overnight at 4°C and brought to body temperature before its application in the microfluidic setup.

**Table 1:** Sequential steps in the preparation of biosimilar mucus (BSM)

Buffer	Dissolving HEPES (1 mM), CaCl_2_ (1.3 mM), MgSO_4_ (1 mM), and NaCl (137 mM) in Milli-Q water and adjusting the pH to 7.4
Isotonic buffer	Dissolving HEPES (1 mM), CaCl_2_ (1.3 mM), and MgSO_4_ (1 mM) in Milli-Q water and adjusting the pH to 7.4
Lipids mixture	Dissolution of PC (0.18% W/V), Cholesterol (0.36% W/V), Tween 80 (0.163% W/V), and Linoleic acid 0.11% W/V) in the isotonic buffer
Carbomer was added to the buffer with magnetic stirring, followed by the incorporation of mucin powder into the mixture using a vortex mixer. Next, the lipid mixture was added, and BSA was incorporated into the mixture with reduced stirring. Finally, the pH of the mixture was adjusted to 7 using a 5M NaOH solution	

The viscosity of mucus in the intestine varies based on the distance from the epithelial layer, the presence of bacteria, and any infections [32–35]. Some studies use low-viscosity mucus mimics [27], while others use high-viscosity models [28] to mimic the intestinal mucus barrier on a chip. To explore rheological effects, two BSM variants were used: BSM-1, which included all components except Carbomer, and BSM-2, which comprised all components, with Carbomer providing elastic properties to the BSM. Carboxylate-modified polystyrene particles, 1 µm in size and exhibiting red fluorescence (with emission and excitation wavelengths of 580 nm and 605 nm, respectively), were diluted in BSM at a 1/20000 ratio for use as markers to extract velocity field and dislodgement rate of mucus.

Earlier studies have often employed water as a surrogate for lumen material [21, 27]. However, water fails to capture the pH, ionic mobility, and viscosity of intestinal physiological fluids. Consequently, research has advocated for the utilization of HBSS buffers [36], bile salts solutions [37, 38], and simulated intestinal fluids [39] due to their closer resemblance to actual intestinal fluids. Given the widespread application and accessibility of HBSS, this study has chosen to use it as the lumen material. Yellow-green carboxylate-modified polystyrene particles, 4.5 µm in size (with emission and excitation wavelengths of 441 nm and 486 nm, respectively), diluted in HBSS at a 1/1000 ratio, were used as marker to extract the velocity field in the lumen material and penetration rate of the particles into the mucus layer.

### On-chip investigation

**Fig. 3:**
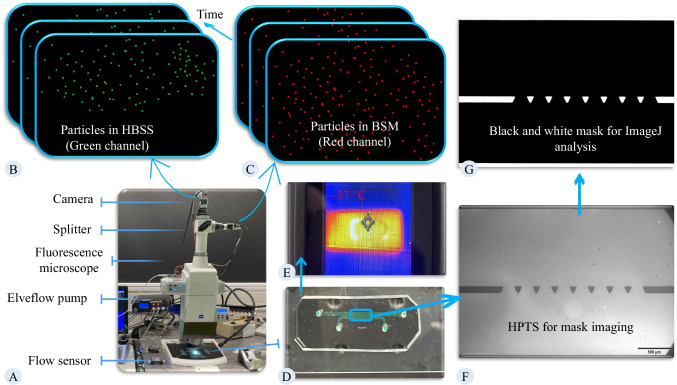
**Experimental setup illustration:** (**A**) Experimental setup comprising a Zeiss microscope for visualization of the fluorescent particles and an Elveflow pump for the injection of the HBSS into the microfluidic chip. (**B**) Time series of captured images aligned to the green channel (HBSS). (**C**) Time series of captured images aligned to the red channel (BSM). (**D**) Representation of the microfluidic chip filled with HPTS. (**E**) Thermal camera image showing the temperature of the mucus inside the chip just before the experiment began. (**F**) Illustration of the microfluidic chip area under microscopic observation using a 1 × lens at 45% zoom, focusing on the region of interest (ROI). This image was captured post-experiment with an HPTS solution injected (panel **D**) to define the ROI. (**G**) Black and white mask used for image processing, derived from thresholding the mask shown in panel **F**

The microfluidic device, visualized under a Zeiss Microscope (Axio Zoom V16) as shown in Fig. 3, was used for the observation of corresponding fluorescent particle movement within both the mucus and HBSS. The fluorescent microscope was equipped with an excitation filter (CHROMA, 59022x) and splitter to enable the detection of two channels (red and green). Each channel consists of a filter (Thorlabs MF525-39 for green and MF630-69 for the red channel) and a camera (Basler acA5472-17μm) to separate and capture the emitted light from fluorescent particles. Considering the variable flow rate of intestinal material due to differing wall movements (segmentation and contraction), the study adopted a uniform inlet velocity of approximately 0.15 mm/s for the lumen channel, as suggested by Palmada et al*.* [40], resulting in a calculated flow rate of 0.054 mL/hr for the lumen material. Initially, the device was saturated with BSM and equilibrated to body temperature, as shown in Fig. 3-E. After positioning the chip under the Zeiss microscope, the outlet of the upper (lumen) channel was immersed in water to keep atmospheric pressure. Subsequent HBSS injection followed the flow rate of 0.05 mL/hr, with the Elveflow flow control pump (OB1 MK4) and Elveflow flow sensors (MFS2) ensuring precise flow rate management. During HBSS injection, which followed BSM injection, the inlet and outlet of the mucus channel were sealed with specialized plugs. Imaging was performed at a frequency of 15 frames per second, using a 1 × lens and 45% zoom to align with the area depicted in Fig. 3-F (region of interest), capturing images at various depths throughout the experiment. Panel B and C of Fig. 3 depict the images captured by cameras aligned for the green and red channels, respectively. At the end of the experiment, to visualize the field of view (2970.20 µm × 1980.13 µm at a resolution of 0.54428 µm) (Fig. 3-F), the HPTS solution (3 mg/mL of 8-hydroxypyrene-1,3,6-trisulfonic acid in water) was injected into the device to take a picture as a mask, helping to identify the solid components of the microfluidic chip. Two experimental series were conducted, one with BSM-1 and the other with BSM-2, under consistent conditions regarding microfluidic design, temperature, pressure, lumen material, particle size, and type. The distinction between the series was the type of BSM used. Each series involved recording red fluorescent particles in BSM and green fluorescent particles in HBSS.

### Image analysis

To determine the velocity field in the mucus and lumen material and penetration of the luminal particles into the mucus layer, images captured during the experiment were analyzed employing ImageJ software (version 1.54f) in conjunction with the TrackMate (version v.7.11.1) plugin [41, 42]. The Laplacian of Gaussian (LoG) detector within TrackMate was utilized for particle detection, effectively identifying potential particle locations by analyzing intensity variations in the images. Following detection, particle tracking was executed using advanced Kalman filter algorithms, also available in TrackMate. These algorithms predict the next position of a particle based on its earlier locations while incorporating measurement uncertainties, thus offering robust tracking capabilities. The LoG detector was configured with a quality threshold of 2 and a radius of 5. Additionally, the parameters of the Kalman filter were set to 80 for the initial research radius, 20 for the search radius, and 5 for the maximum frame gap (more details at [41, 42]). Dark-field imaging mode was used to ensure particles were visible in the recorded images. A mask, as shown in Fig. 3-G, was aligned for the image series, showing the solid (PDMS) parts of the images. The mask was generated by applying the Intermodes algorithm in ImageJ to the image in panel F of Fig. 3, with threshold boundaries set at 75 and 255. Once the particle trajectories were obtained, the average velocity and location of tracks (available within TrackMate) were analyzed to elucidate the underlying phenomena, providing insights into the movement and behavior of the mucus layer under the influence of shear stress. The data obtained from ImageJ were initially in inches and frames, which were then converted into micrometers and seconds using the imaging resolution (0.54428 µm) and frame rate (15 frames per second).

### Rheology of BSM

For the rheological measurements of mucus, the study employed a Discovery DHR-2 Rheometer equipped with a cone and plate geometry, featuring a cone angle of 1° and a diameter of 40 mm. Throughout the experiments, the plate temperature was maintained at 37°C to simulate body temperature conditions, and dehydration of the sample was prevented by applying low-viscosity silicone oil (5 mPa S at 25°C) into a custom-made solvent trap. The flow sweep test was performed at a shear rate range of 0.1–100 s⁻^1^ to analyze viscosity changes with shear rate. Oscillatory shear tests, specifically frequency sweep tests, were conducted to examine the linear viscoelastic region (LVR) of the samples across a frequency range of 0.1–100 rad/s at 3% strain. This frequency range is essential for evaluating the stability of the mucus structure and its viscoelastic properties [43]. Rheological data, including the storage modulus (G′), loss modulus (G″), and viscosity, were obtained using TA Instruments Rheology Advantage software.

### Contact angel measurement

To measure the contact angle of BSM and HBSS on PDMS, the setup illustrated in Panel G of Fig. 5 was used. As a reference, the contact angle of the DIW on the PDMS was measured. PDMS generally has a hydrophobic surface, but the degree of hydrophobicity depends on the time it is kept in the oven after treating it with corona discharge for bonding the glass slide. For all the experiments and measurements in this study, the PDMS was kept in the oven for 2 h before measurements. For all measurements, 10 µL of the sample was placed on the substrate and the resulting droplet was photographed with a camera. Measurements were done at atmospheric pressure, with the temperature of the DIW at 20 °C and the BSM and HBSS at 37 °C. Captured images were analysed using the LBADSA plugin [44] of ImageJ.

### In silico study

**Fig. 4:**
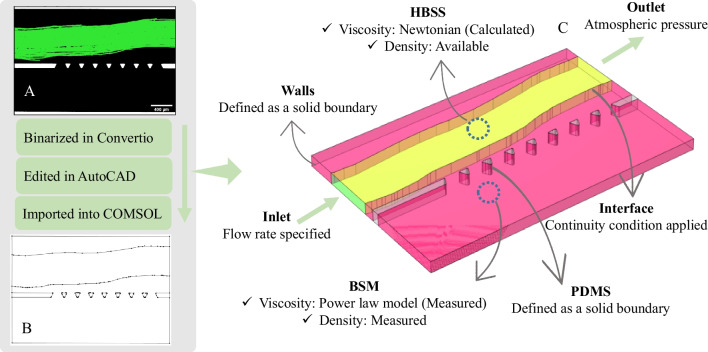
**Sequential steps used in the *in-silico* study:** (**A**) A representative image captured during the experiment, showing the green region representing HBSS flow and the black region representing BSM-2. This image was used to generate the geometry for numerical simulations of the experiment conducted with BSM-2. (**B**) A binarized version of the image in panel A, processed using Convertio and further edited in AutoCAD before being imported into the COMSOL software. (**C**) The final 3D geometry used for numerical simulations, incorporating the corresponding boundary conditions. The corresponding 3D geometry for the numerical simulation of the experiment conducted with BSM-1 was also generated following the same procedure, using the image shown in panel A of Fig. 12

This section aims to simulate the flow field within the HBSS and mucus layer using mathematical models. Investigating the impact of induced shear stress by the flowing lumen material on the mucus layer necessitates the simulation of the velocity field within the mucus layer. To determine the velocity and pressure field, continuity (1) and momentum (2) equations are concurrently solved.

1 $$\frac{\partial \rho }{\partial {\varvec{t}}}+\nabla .\left(\rho {\varvec{U}}\right)=0$$

2 $$\rho \left({\varvec{U}}.\nabla \right){\varvec{U}}=\nabla .\left[-P+2\mu \left(\nabla {\varvec{U}}+{\left(\nabla {\varvec{U}}\right)}^{{\varvec{T}}}\right)\right]$$

here ρ is the fluid density, *μ* is the viscosity, *P* is the pressure, and *U* is the velocity field. These equations apply to laminar flow. With reported Reynolds numbers for flow in the intestine being less than 200, the flow regime can be considered laminar [45]. To simulate the microfluidic observation, captured images from the experimentation phase were processed through the Convertio platform, an online image converter (Fig. 4-A). The output from Convertio was a DXF file, which underwent further refinement in AutoCAD software to ensure a smooth drawing and maintain the same scale as the microfluidic chip. This refined version was then saved as a mask for geometry (Fig. 4-B). Subsequently, the mask was imported into COMSOL software and extruded to generate the 3D geometry necessary for numerical simulation (Fig. 4-C).

The accuracy of numerical methods strongly depends on the simulation parameters. Using these (physicochemical) parameters, mathematical models (governing equations) can be adapted to special problems. The parameters used in the simulation and the method of selecting these parameters are provided in Table 2. Boundary conditions are practically essential for defining a problem. The solution to the differential equation should satisfy the boundary conditions. Simulations are performed at atmospheric pressure. The flow of lumen material induces shear to mucus and forces the mucus to move. Boundary conditions are defined based on the experimental conditions, including the flow rate for the inlet of the lumen channel, atmospheric pressure at the outlet, and no-slip velocity at the PDMS walls (Fig. 4-C). The computations were conducted using COMSOL Multiphysics 6.1 on a Microsoft Windows 10 Enterprise system. The machine boasted 150 GB of memory, and throughout the simulations, the maximum memory usage reached 8 GB.

**Table 2:** Physicochemical properties were used in numerical simulations

HBSS	Density	1085 [kg.m.^−3^]
	Viscosity	To calculate the viscosity of the HBSS solution, the Falkenhagen [46] and Jones and Dole [47] relations were used (3) $\frac{\mu }{{\mu }_{0}}=1+\frac{0.517{Z}^{2}}{L{\mu }_{0}\sqrt{{\varepsilon }_{0}T}}\sqrt{C}$ (3) Where *μ* is the viscosity of the solution, *μ*_*0*_ is the viscosity of the solvent (1e-3 [Pa.s]), *C* is the concentration of the solute (0.16 M), *ε*_*0*_ is the dielectric constant of the solvent (78.4), *T* is the absolute temperature (310 [K]), *L* is the ionic mobility at infinite dilution (average mobility of solutes in the HBSS ≈ 3 × 10^–3^ [m^2^/ (V·s)]), and *Z* is the ionic valences (average ionic valences of solutes in the HBSS ≈ 1). Using this relation, the approximate viscosity of HBSS was calculated as 0.0261 [Pa.s]
BSM-1 & BSM-2	Density	The density of the biosimilar mucus was measured by weighing 1 mL of the BSM.
	Viscosity	The viscosity of the BSM was defined using the power-law model (4) $\mu =K{\gamma }^{\text{n}-1}$ (4) Where *μ* is the viscosity, *K* is the consistency index, *n* is the flow behavior index, and *γ* is the shear rate. *K* and n are calculated by fitting the rheological data (measured in the Rheology of BSM section) on the power-law model

## Results

The results section begins with the physicochemical characterization of BSM and HBSS, providing essential information for designing a microfluidic approach and conducting a numerical study of the mucus barrier. The Mucus on a chip device optimization section evaluates the current microfluidic approach to studying the mucus barrier, highlights its limitations, and introduces a method that enables the study of the mucus barrier under prolonged exposure to lumen materials and fluidic lumen conditions. The Intestinal mucus barrier on a chip section examines the performance of the optimized microfluidic approach for studying the mucus barrier, addressing both low- and high-viscosity mucus models. The Velocity field section investigates the velocity distribution in the lumen and mucus channel, analyzing the influence of viscosity on the velocity field. Finally, the Numerical simulation section presents a numerical simulation approach validated by experimental observations.

### Physicochemical characteristics of BSM and HBSS

The study of the intestinal mucus barrier using a microfluidic approach requires consideration of the physicochemical phenomena dominant on a small scale, such as viscous forces and capillary forces. Moreover, to conduct numerical simulations, parameters such as the constitutive equation for viscosity and density of the BSM and HBSS are required. This section aims to conduct these measurements for the BSM and HBSS.

**Fig. 5:**
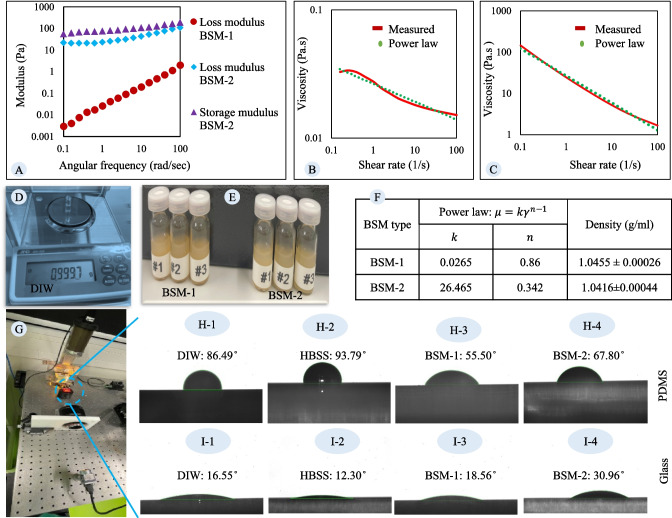
**Physicochemical characterization of BSM and HBSS for microfluidic design and numerical simulations:** (**A**) Loss and storage moduli of BSM-1 and BSM-2 plotted against angular frequency (0.1–100 rad/s), measured using oscillatory shear tests at 37 °C. BSM-2, containing Carbomer, demonstrates significantly higher elastic properties and a dominant storage modulus, indicating a structured mucus compared to BSM-1. (**B**) Flow sweep results for BSM-1, showing the viscosity variation with shear rate (0.1–100 s⁻^1^) at 37 °C, with a power law model fitted (parameters: consistency = 0.0265, flow behavior index = 0.86, R^2^ = 0.9647). (**C**) Flow sweep results for BSM-2, showing a more pronounced shear-thinning behavior with fitted power law parameters (consistency = 26.465, flow behavior index = 0.342, R^2^ = 0.9928). (**D**) Validation of the density measurement methodology using deionized water (DIW) at 20 °C, showing excellent agreement with the literature value (0.9997 g/mL vs. 0.9982 g/mL). (**E**) Density measurements of BSM-1 (1.0455 ± 0.00026 g/mL) and BSM-2 (1.0416 ± 0.00044 g/mL) were performed in triplicate (*n* = 3). (**F**) Summary of the power law model parameters for viscosity obtained from panels B and C, and the density results from panel E, presented as mean ± standard deviation. (**G**) Schematic representation of the contact angle measurement setup, including the light source, substrate holder (PDMS or glass slide), lens, and camera. Droplets (10 µL) of DIW, HBSS, BSM-1, and BSM-2 were imaged after substrate preparation (PDMS baked at 60 °C for 2 h). (**H**) Images of droplets on PDMS: (H-1) DIW, (H-2) HBSS, (H-3) BSM-1, and (H-4) BSM-2, with corresponding contact angle measurements displayed. The results indicate that BSM exhibits better wettability on PDMS compared to HBSS, highlighting its relevance in microfluidic device design. (**I**) Images of droplets on glass slides: (I-1) DIW, (I-2) HBSS, (I-3) BSM-1, and (I-4) BSM-2, with corresponding contact angle measurements displayed

Figure 5-A displays the loss and storage moduli versus angular frequency for both BSM-1 and BSM-2. BSM-2's formulation, which includes Carbomer, presents elastic properties, resulting in a loss modulus 100 times greater than BSM-1. Furthermore, BSM-2 exhibits a dominant storage modulus, indicating a structured mucus.

The constitutive relation for viscosity can be obtained by fitting mathematical models to the viscosity variation versus shear rate. Panels B and C of Fig. 5 show the results of the flow sweep measurement for the BSM-1 and BSM-2, respectively. To provide a constitutive equation, a power law model was fitted using the Power Trendline feature in Microsoft Excel (panel F of Fig. 5). Parameters of the power law model for viscosity of BSM-2 are calculated as 26.465 and 0.342 for consistency and flow behaviour index, respectively (panel C of Fig. 5) with an R-squared value of 0.9928. The parameters of the power law model for the viscosity of BSM-1 were calculated as 0.0265 for consistency and 0.86 for the flow behaviour index, as shown in panel B of Fig. 5. The R-squared value of the fitting is 0.9647. The flow behaviour index (n < 1) exhibited by the BSM-1 and BSM-2 indicates the pseudoplastic or shear-thinning nature, implying that apparent viscosity decreases at higher shear rates. Rheology measurements conducted on mucus from various animal sources further corroborate the shear-thinning behaviour of mucus [31, 48–50].

The density of the BSM was measured by weighing 1 mL of the sample. As proof of the methodology, the density of the DIW was measured at 0.9997 g/mL (Fig. 5-D). Measurements were performed at atmospheric pressure and a temperature of 20 °C. Under these conditions, the density of the DIW is 0.9982 g/mL [51]. Using the same method, the density of BSM-2 was measured as 1.0416 ± 0.00044 g/mL, and the density of BSM-1 was measured as 1.0455 ± 0.00026 g/mL (Fig. 5-E).

Capillary forces are vital to consider in microfluidic devices. To gain insight into the effect of these forces, the contact angles of the BSM and HBSS on PDMS and glass slides were measured. Panels H-1, H-2, H-3, and H-4 show droplets of DIW, HBSS, BSM-1, and BSM-2, respectively. Results show that the PDMS prefers BSM more than HBSS, and these factors should be considered when designing microfluidic devices. Panels I-1, I-2, I-3, and I-4 show droplets of DIW, HBSS, BSM-1, and BSM-2 on the glass slide, respectively. Surface tension measurements of BSM and DIW further confirm the differences in the contact angle between BSM, HBSS, and DIW [52]. The glass slide has a hydrophilic surface, and as can be seen from the images, water prefers the surface more than BSM. The contact angle of each sample on PDMS and the glass slide is reported in images from panels H and I, respectively. Besides the importance of considering these forces for designing microfluidic devices, it is essential to have this information for numerical simulations.

### Mucus on a chip device optimization

**Fig. 6:**
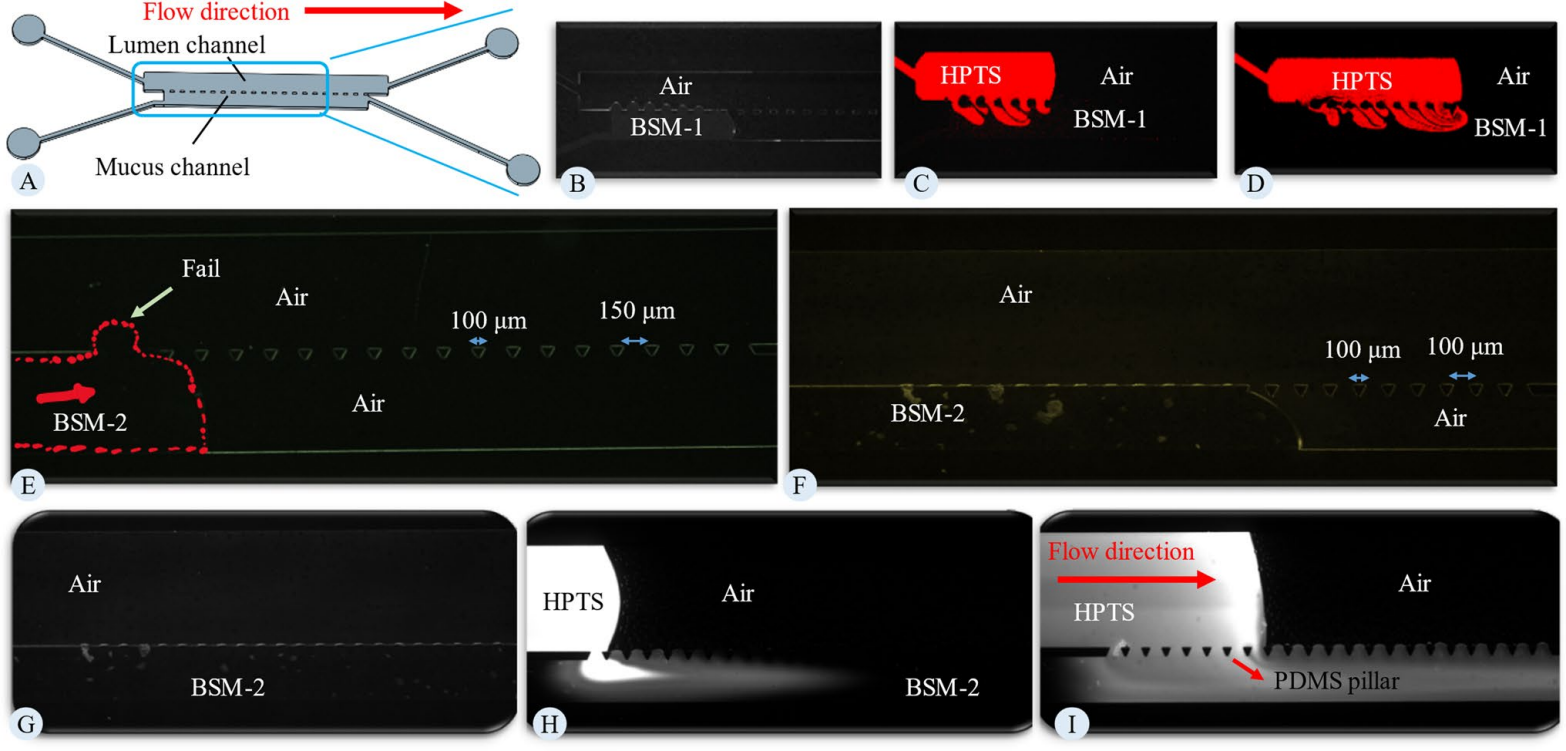
**Microfluidic Design Evolution and Injection Procedure:** (**A**) Microfluidic design to mimic the study by Jia et al. [27]. This design consists of two parallel channels: one for mucus and one for lumen material, separated by interfacing pillars. The upper channel is designated for the lumen, and the lower one is for mucus. The interface pillars measure 200 µm on the large side, 100 µm on the small side, and are spaced 300 µm apart. (**B**) Injection of BSM-1 into the microfluidic chip shown in panel A. (**C** and **D**) Injection of the HBSS solution into the model. HPTS was added to the HBSS to make it fluorescent. Captured images were analyzed in ImageJ, with red color indicating HBSS. (**E**) Injection of BSM-2 into the mucus channel. For this design, the pillar configuration is as follows: the pillars are trapezoidal in shape, with a larger side measuring 100 µm, a smaller side of 20 µm, and spaced 150 µm apart. (**F**) Injection of BSM-2 into the microfluidics, which utilizes pillars of the same dimensions as in panel **E** but with a reduced distance between them, set at 100 µm. (**G**) Microfluidic design similar to that in panel **F**, with BSM-2 injected into the mucus channel. (**H** and **I**) Injection of the HPTS solution into the lumen channel, where the flow demonstrates a preference for the mucus channel over the lumen channel. In this experiment, HPTS is shown in white

In this section, the objective is to develop a microfluidic approach to address the limitations of previous microfluidic devices. To do this, we first conducted experiments under the same conditions as the study by Jia et al. [27] and Wright et al. [28]. They used a microfluidic device with two parallel channels separated by pillars to confine mucus and lumen material. Mucus was injected into the lower channel, while the lumen material was injected from the top channel. We performed a similar experiment using the microfluidic device depicted in panel A of Fig. 6. For this experiment, we used BSM-1, similar to the one used in Jia et al.’s study [27]. The mucus was injected into the bottom channel. The challenge here is to keep the mucus confined to the lower channel, a limitation also noted in previous studies of proposed micromodels. Continuing the injection of BSM-1, as shown in panel A, will result in the failure of capillary forces and cause BSM to flow into the lumen channel. If the injection of BSM is halted at this point and the injection of the lumen material is started, the lumen material (in this case, HBSS with HPTS) will push the mucus layer (panel B of Fig. 6). This pattern was also observed in Jia et al.'s study [27]. The reason for this observation is the different contact angles of HBSS and BSM-1, as shown in panel H of Fig. 5. PDMS interacts more favorably with BSM than with HBSS, resulting in less resistance to flow in the mucus channel compared to the HBSS channel. Consequently, the mucus is replaced by HBSS instead of forming an interface that would allow for observation of penetration (panels C and D of Fig. 6). This strategy is unsuitable for measuring permeability. Therefore, the first solution is to address the challenge of the interfacing pillars to successfully retain the mucus.

Panel E of Fig. 6 shows the injection of BSM into the mucus channel and the formation of a droplet between the interfacing pillars. This droplet demonstrates how the pillars fail to keep the mucus confined to the proper channel. Considering the dominant forces, we have a pressure gradient supplied by the pump, viscous forces resulting from applied shear stress by the walls of the channels, and capillary forces. To ensure that the flow remains within the mucus channel without leaking into the lumen channel, the hydraulic resistance of the mucus channel must be less than the hydraulic resistance of the space between the pillars. We tested several distances and configurations of the pillars and found that the configuration shown in panel F of Fig. 6 is optimal for retaining mucus in the channel. These pillars successfully keep the mucus in the proper channel for both BSM-1 and BSM-2.

Panel G of Fig. 6 shows the optimized micromodel, which is the same design as shown in panel A but with optimized pillars in panel F. In this experiment, BSM-2, similar to the mucus model used in the study by Wright et al. [28], was injected into the mucus channel. As shown in the figure, the mucus successfully filled the entire mucus channel without leakage. Following this, HBSS was injected from the lumen channel. As seen in panels H and I of Fig. 6, the flow of HBSS prefers the mucus channel over the lumen channel. This observation is consistent with the study by Wright et al. [28]. The reason for this preference is the buildup of capillary pressure. Examining the interface of HBSS and air in panel H of Fig. 6, it is evident that the pressure on the air side is higher than on the HBSS side. To continue the flow in the lumen channel, the pressure on the HBSS side must exceed that on the air side. An increase in pressure on the HBSS side causes flow into the mucus channel, indicating that the resistance in the mucus channel is lower than in the lumen channel, as there are no capillary forces in the mucus channel. We also attempted to close the mucus channel inlets and outlets after injecting the mucus to prevent issues with flow in the mucus channel. In this case, the pressure in the HBSS increased, and since it is connected to the mucus through the first set of pillars, the pillars at the end of the channel failed to retain the mucus. This suggests that the pressure buildup in the HBSS was greater than the capillary pressure provided by the pillars to keep the mucus in the lower channel.

We tried numerous configurations to address this issue, including altering the hydrophobicity of the PDMS, testing different injection scenarios (parallel and countercurrent), varying the flow rates for injection, using pillars with a short distance from each other (30 µm), and even simultaneously injecting BSM and HBSS. However, none of these approaches were successful. Subsequently, we decided to initially saturate the model with mucus and then seal the mucus channel's inlet and outlet. We then commenced the injection of the lumen material from the lumen inlet. This approach successfully established a stable scenario for maintaining both mucus and lumen material adjacent to each other for an extended period. The following section describes the application of this approach to study the mucus barrier on a chip.

### Intestinal mucus barrier on a chip

The previous section investigated several approaches to studying the mucus barrier on a chip and identified the use of the saturated micromodel as the solution. All subsequent experiments in this work were conducted using saturated models with the design, experimental techniques, and materials described in the methodology section. This section outlines observations regarding particles movement within the mucus layer and lumen material. The experiments are divided into two series: one involving BSM-1 and the other involving BSM-2. Each experiment includes two sets of images: one for the red channel (representing BSM) and the other for the green channel (representing HBSS). At the onset of the experiment, the chip is saturated with BSM, resulting in the absence of green particles within the region of interest (ROI). However, as the experiment progresses, green particles appear, indicating the influx of HBSS. Red particles may be found in both HBSS and BSM, due to the HBSS flow dissolving some of the BSM containing red particles. Images from each channel (red and green) are analysed separately, and trajectories are examined to understand the interactions between the mucus and lumen material.

**Fig. 7:**
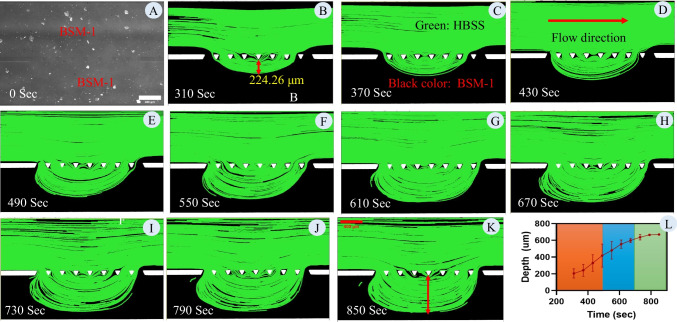
**Establishment and analysis of HBSS flow into BSM-1 using a saturated micromodel**: (**A**) Micromodel saturated with BSM-1 before introducing HBSS, demonstrating the region of interest (ROI). (**B-K**) Temporal evolution of HBSS flow into the BSM-1 layer, visualized through 60-s interval snapshots from 310 to 850 s. The green areas represent the penetration and flow of HBSS, while the black areas indicate the remaining BSM-1. A clear interface forms and progressively advances as HBSS replaces BSM-1. The initial rapid penetration phase reflects high HBSS velocity through limited flow pathways, followed by a deceleration as the interface stabilizes and mucus replacement slows. Images were analyzed using ImageJ, with the green channel used to map HBSS regions and trajectories. (**L**) Quantification of the penetration depth of HBSS into BSM-1, measured as the distance from the interface pillar (as shown in Panel **B** and **K**). The penetration depth is segmented into three distinct phases: initial rapid penetration, intermediate deceleration, and final stabilization of the HBSS-BSM interface. Effective diffusion coefficients (D = L^2^/t) for these phases are calculated as 415.21 µm^2^/s, 31.91 µm^2^/s, and 1.78 µm^2^/s, respectively. The final phase aligns with literature-reported values for mucus diffusion coefficients, emphasizing the relevance of the established interface for studying marker transport within mucus

Figure 7 shows the results of the experiment with BSM-1. Panel A depicts the design saturated with BSM-1. Following the saturation and the closing of the inlet and outlet of the mucus channel, the lumen material was injected through the lumen inlet. Panels B to K illustrate the expansion of the region where HBSS flows and its temporal evolution. Images taken during the experiment were analyzed using ImageJ. Green areas represent the region of the HBSS, while black areas indicate BSM-1. As seen from the figure, the flow of HBSS through BSM-1 creates a clear interface between BSM and HBSS. Initially, the penetration into the BSM is rapid. However, after establishing a stable interface, the interface can be tracked to measure penetration into the mucus layer. To quantify the penetration into the mucus layer, panel L shows the penetration into the mucus layer. This expansion is quantified as a depth from the interface pillar, as depicted in Panel B. Initially, the observed rapid growth primarily signifies the replacement of the mucus layer with HBSS. This accelerated expansion can be attributed to the high velocity during the initial stages, owing to the limited area available for HBSS flow. Subsequently, the rate of depth increase decreases due to the lower velocity and consequently reduced induced shear on the BMS-1. However, in the later stages (after 730 s), this growth decelerates further, signaling the establishment of the interface between BSM-1 and HBSS. This stage can serve as a reference point for measuring penetration into the mucus layer. Defining the effective diffusion coefficient as D = L^2^t^−1^, where L represents the penetration length and t denotes time. In panel L, the effective diffusion coefficients for three segments of the graph are calculated as 415.21, 31.91, and 1.78 µm^2^/sec, respectively. Notably, the effective diffusion coefficients for the mucus from the literature are in the same order of magnitude as the value calculated for the last segment of the graph [37, 38, 53]. It is crucial to consider the penetration process following the establishment of an interface between the luminal material and the mucus layer. Determining the establishment of this interface relies on factors such as microfluidic design, mucus viscosity, luminal material viscosity, and the flow rate or velocity of the luminal material.

**Fig. 8:**
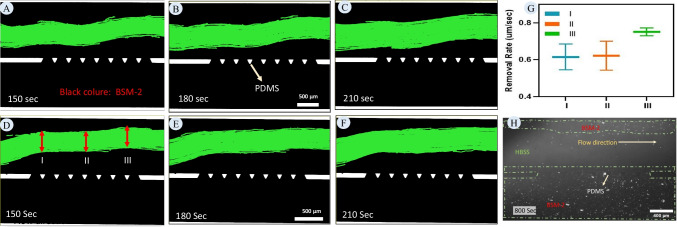
**Establishment of the flow of HBSS into BSM-2:** (**A-C**) Time series of HBSS flow through BSM-2, captured at 150, 180, and 210 s, respectively. The green areas represent regions occupied by HBSS, while the black areas indicate BSM-2. The interface between the green and black regions marks the boundary between HBSS and BSM-2. Unlike BSM-1, where mucus is removed by HBSS flow, the high viscosity and elastic properties of BSM-2 result in the formation of a stable channel that HBSS continues to follow. (**D-F**) Results from repeated experiments, showing consistent patterns of HBSS penetration and channel formation through BSM-2. These images demonstrate the robustness of the microfluidic approach in tracking HBSS flow dynamics, regardless of initial pattern variations. (**G**) Quantitative analysis of HBSS flow thickness over time, measured at three distinct points (I, II, and III) identified in panel **D**. The thickness of the HBSS flow region increases as mucus is gradually removed. The data represent the mean values from three independent experiments (*n* = 3), with error bars indicating standard deviation. (**H**) Final state of the flushing experiment performed at a higher velocity (0.2 mL/hr), showing how increased flow velocity accelerates mucus removal and further stabilizes the interface between HBSS and BSM-2

The preceding sections demonstrated the application of the proposed microfluidic approach to measure penetration into low-viscosity mucus models. In this section, the objective is to examine the same approach using BSM-2 as a high-viscosity mucus model. This is achieved by analysing the trajectories of green particles in experiments conducted with BSM-2. Initially, the model is saturated with BSM-2, and subsequently, HBSS is injected into the model through the lumen channel. Panels A to C of Fig. 8 show the flow of HBSS through BSM-2. Unlike with BSM-1, where mucus is removed, the flow of HBSS creates a channel through BSM-2 and continues to follow that channel. This behaviour is due to the high viscosity and elastic properties of BSM-2, which offer more resistance to the flow. Panels D to F illustrate the same phenomenon through repeated experiments with different patterns. Regardless of the initial pattern, the interface can be tracked to calculate penetration into BSM-2. The approach used in the previous section for BSM-1 can be applied to quantify the penetration into BSM-2. Since BSM-2 offers significant resistance to flow, the injection velocity can be reduced once the interface is established to maintain stability. Alternatively, it is possible to continue flushing the system at high velocity (as shown in panel H) to remove mucus from the lumen channel, forming the interface at the pillars and tracking the penetration thereafter.

Besides using the proposed microfluidic approach to measure the permeability of the mucus layer, it could also be used to monitor the removal of the mucus layer. Over time, parts of the mucus layer dissolve into the lumen material, a process facilitated by the induced shear from the flowing lumen material. Figure 8, panels D to F, show the expansion of the HBSS flow area over time, corresponding to the area of mucus removed by HBSS. Figure 8-G provides insight into the removal rate of BSM-2 over time, indicating the speed at which HBSS can clear BSM, a process heavily dependent on the velocity of HBSS.

### Velocity field

**Fig. 9:**
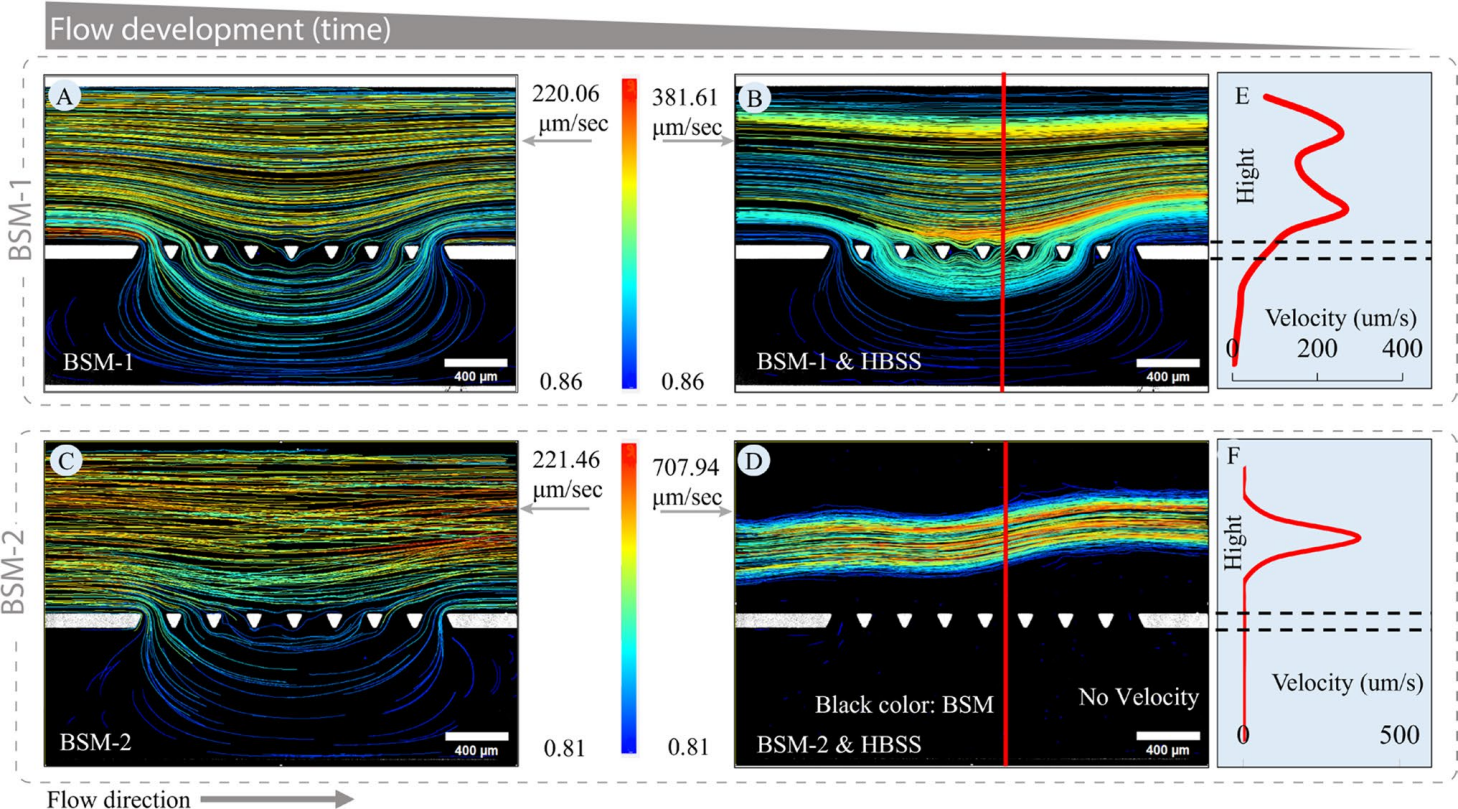
**Rheology's impact on mucus movement:** (**A-D**) Display the average velocity of particle tracks from ImageJ analysis for BSM-1 and BSM-2 during the first and third 60-s intervals of the experiment, respectively. Each track color represents the average velocity of particles along that track. Tracking is performed for the red particles dispersed in the BSM-1 and BSM-2. The tracks of the green particles in the HBSS are not considered in this part. Panels **A** and **C** show the velocity of the BSM-1 and BSM-2 during the first 60 s when only mucus is in the ROI. Panels **B** and **D** show the velocity during the third 60 s when HBSS is in the ROI. Since HBSS dissolves the BSM and contains red particles, red particles are also present within the flow of the HBSS. Black areas in the figures represent stagnant mucus, and the tracks indicate where there is movement in the mucus. Panels **E** and **F** show the velocity profile along the cross-section of the channel (red lines in panels **B** and **D**) for BSM-1 and BSM-2, respectively

The previous section demonstrated how to use the proposed microfluidic approach for measuring the permeability and removal rate of the mucus. In this section, by analysing the velocity profile, the aim is to support the findings of the previous section and also provide observations for the validation of numerical methods. To measure the velocity variation in the mucus and lumen channel for both BSM-1 and BSM-2, an analysis was conducted focusing on the behaviour of red particles within each experiment series. Figure 9 presents the tracking of red fluorescent particles within two types of BSM over two intervals: the first 60 s and a subsequent 60-s period later in the experiment. The average velocity of the particles' tracks is plotted, with each color standing for the velocity and indicating the average velocity of particles along that track. This method estimates the velocity distribution within different regions of the mucus layer. The flow rate was maintained at 0.05 ml/hr for both BSM-1 and BSM-2 experiments.

For BSM-1, Fig. 9 panels A and B show the average velocity of tracks during the first and third 60-s intervals, respectively. Initially, the flow within the ROI is solely due to BSM, allowing for the establishment of flow in both the mucus layer and the lumen channel. However, as HBSS is introduced, BSM begins to dissolve into it, and red particles appear within the HBSS flow regions. Despite this, flow within the mucus layer persists, as seen in Fig. 9-B, due to BSM-1's rheological properties. In contrast, panels C and D of Fig. 9 illustrate the average velocity of tracks for BSM-2. Similar to BSM-1, the initial 60 s show flow establishment. While, in the subsequent interval, the flow pattern changes markedly as HBSS becomes the dominant flowing medium, creating pathways through the mucus layer. Due to BSM-2's higher viscosity and elastic modulus, it presents greater resistance than HBSS, leading to negligible flow in the mucus channel, as depicted in Fig. 9-D.

Rheological assessments (panel A of Fig. 5) validate the on-chip observations. These rheological findings are consistent with the microfluidic experimental observations, supporting the role of BSM rheology. Panels E and F in Fig. 9 represent the velocity profile along the cross-section (highlighted in red in panels B and D) of the chip. These velocities are calculated by averaging along the line, revealing differences in velocity order between the two BSM variants.

**Fig. 10:**
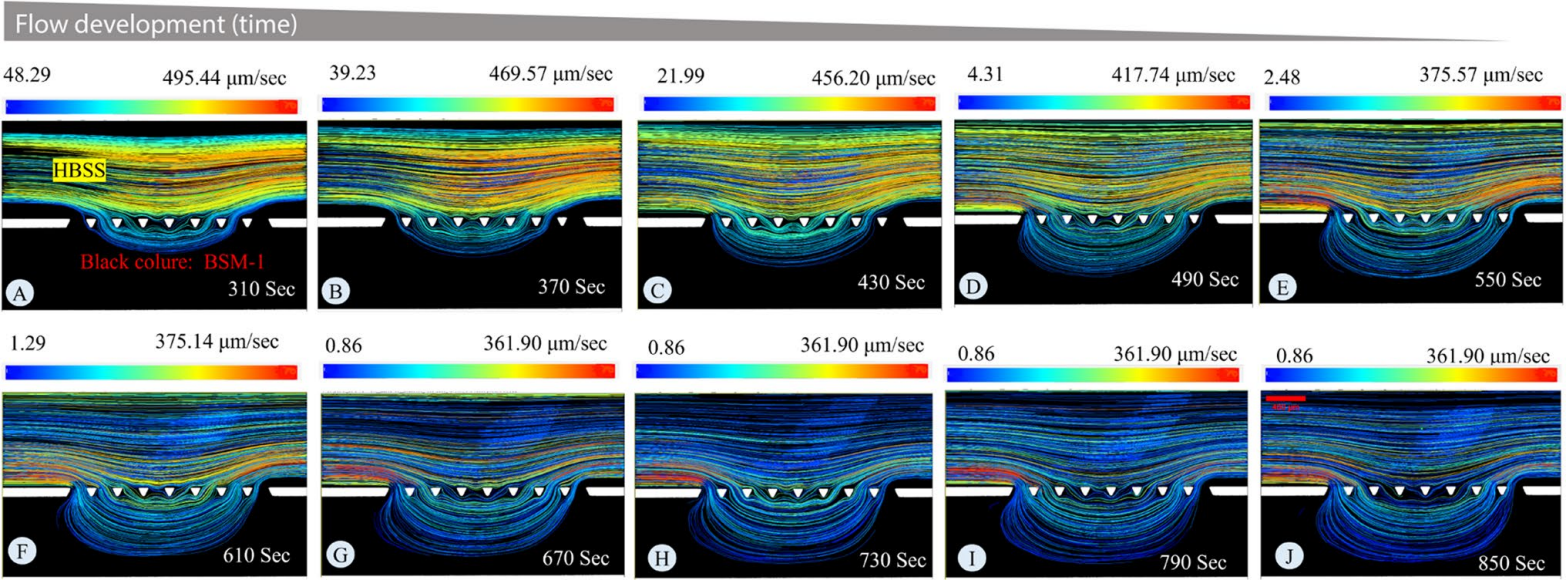
**Velocity distribution in HBSS for experiment with BSM-1:** The trajectory of green particles in the BSM-1 experiment is meticulously scrutinized, particularly focusing on their average velocity over 60-s intervals. Panels **A** to **J** provide comprehensive insights into the establishment of the velocity field. In the initial panels, the velocity is high due to the restricted flow area for the HBSS, as confirmed by the continuity equation, which correlates flow area with velocity. As the flow area increases, the velocity decreases. Beyond panel H, there are no significant changes in the velocity profile

Figure 9 showed the velocity variation in the mucus. In this section, the objective is to evaluate the velocity field in the HBSS for experiments with BSM-1. As discussed in the Intestinal mucus barrier on a chip section, to evaluate penetration into the low-viscosity mucus model, the measurement should be taken after establishing a stable interface between the mucus and the lumen material. To demonstrate the establishment of the interface and velocity variation over time in the HBSS, the focus is on analysing the trajectory of green particles suspended in HBSS in experiments conducted with BSM-1, as depicted in Fig. 10. This figure shows the particle tracks over 60-s intervals at different time points. Panels A to J illustrate the average velocity of the streamlines and their evolution over time. As the flow is established, the velocity of the particles decreases as the flow area increases, supported by the continuity equation. By comparing the velocity profiles in panels H, I, and J, it is evident that there are no significant changes in the velocity profile. This indicates the time or area where flow is established.

### Numerical simulation

Numerical simulation is a valuable tool for predicting penetration into the mucus layer and investigating the removal of the mucus layer. However, these techniques need to be verified against experimental observations. In our previous study [19], we demonstrated the power of CFD in evaluating penetration into the mucus layer. However, due to the lack of physicochemical parameters for intestinal mucus, we used approximate parameters from respiratory tract mucus. In this section, the aim is to use the parameters provided in the Physicochemical characteristics of BSM and HBSS section to develop a numerical approach and validate it against experimental observations.

**Fig. 11:**
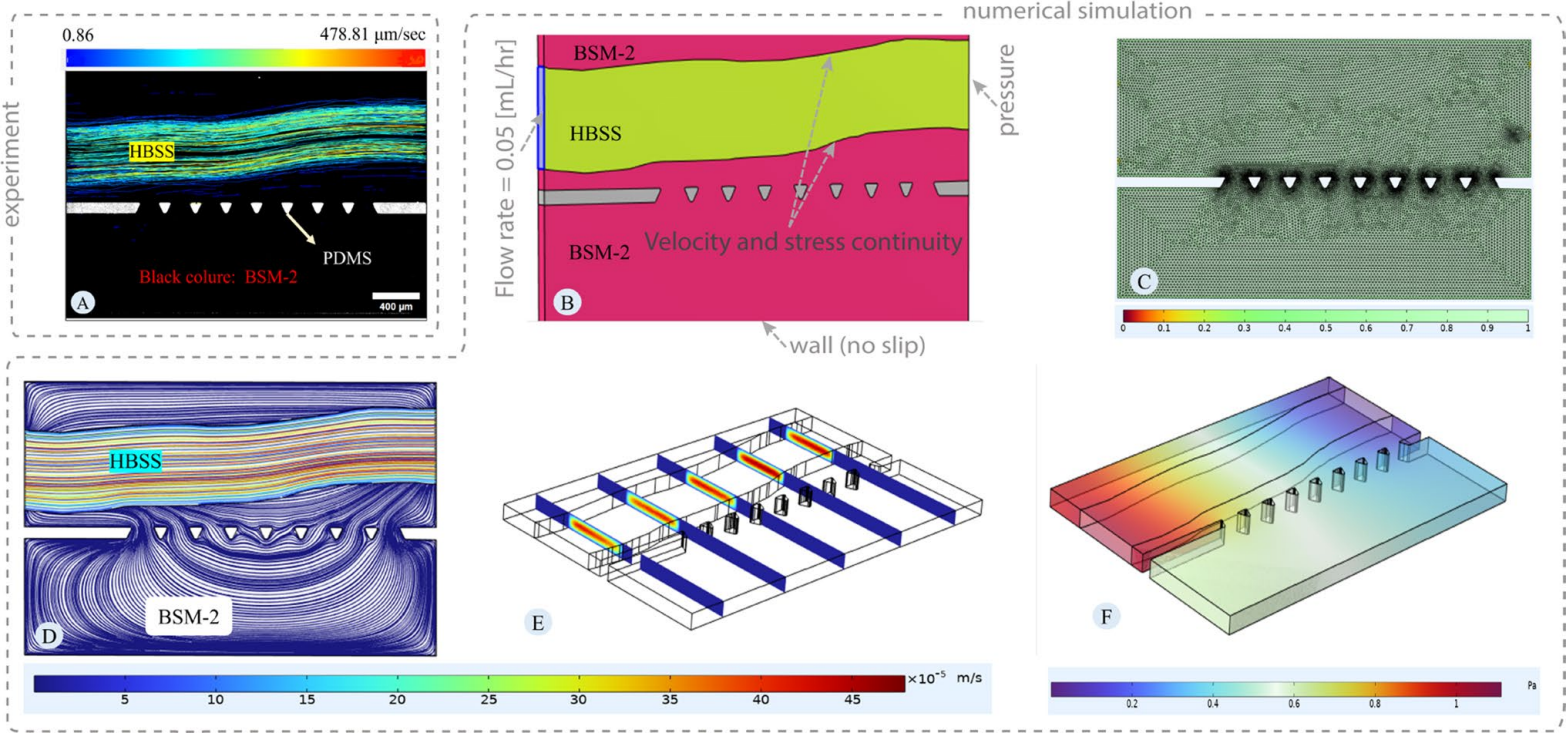
**Velocity field for BSM-2:** Panel **A** shows the velocity distribution by tracking the green and red particles. Tracking is done for the experiment with BSM-2 from 240 to 300 s. Panel **B** presents the 3D geometry utilized for simulation and boundary conditions. The image in Panel **A** is binarized using the Convertio platform and then imported into AutoCAD to scale and fine-tune it. The image is subsequently imported into COMSOL software to create the 3D geometry by extruding the 2D map. Panel **B** shows the 3D geometry in COMSOL software, based on the image in Panel **A**, representing the microfluidic device during the experiment. The pink areas in Panel **B** correspond to the black areas in Panel **A**, representing BSM-2, and the yellow areas in Panel **B** correspond to the green areas in Panel **A**, representing HBSS. Panel **C** shows the mesh quality plot. Panel **D** displays the velocity streamlines, indicating the flow pattern and velocity magnitude. Panel **E** illustrates the velocity profile over cut planes, showing the variation of velocity in 3D orientation. Panel **F** shows the pressure distribution

Panels A Fig. 11 displays the average velocity of tracks during the BSM-2 experiment, encompassing particle tracks from 240 to 300 s. Panel A delineates the trajectory of the green and red particles, illustrating the region where HBSS and mucus flows and assessing velocity profiles within this area. This measurement was selected as a case study for the validation of the modelling approach. Panel B shows the extracted 3D geometry based on the image in Panel A. Numerical simulation was performed using COMSOL software. Simulation is done using the equation provided in the In silico study section. BSM and HBSS were considered incompressible fluids. Fluid flow equations were solved in stationary mode. A physics-based meshing technique was utilized in COMSOL, which implies fine meshing near the boundary for fluid flow equations due to the high gradient (Fig. 11-C). Verification of numerical mesh quality was conducted using COMSOL's average and minimum mesh quality. Panel B of Fig. 11 depicts the 3D geometry and boundary condition used for simulation to mimic the exact condition from the experiment.

Panel E of Fig. 11 illustrates the variation in velocity across the cut plane. Notably, the flow velocity within the HBSS is substantial due to its significantly lower apparent viscosity compared to the BSM (approximately 1000 times lower). Near the walls, velocity diminishes to zero due to the no-slip boundary condition, while it peaks at the center of the channel, away from the walls. In Panel D, the streamlines in the model are depicted. These streamlines encompass velocities both near the walls and in the center of the channel, resulting in non-smooth coloring that represents velocity variations along the streamlines. Streamlines in the numerical simulation show the pattern of the flow within the HBSS and BSM, which is the same as the tracking of the particles from the in-vitro investigation (panel A). Pressure distribution for maintaining the flow is provided in Panel F.

**Fig. 12:**
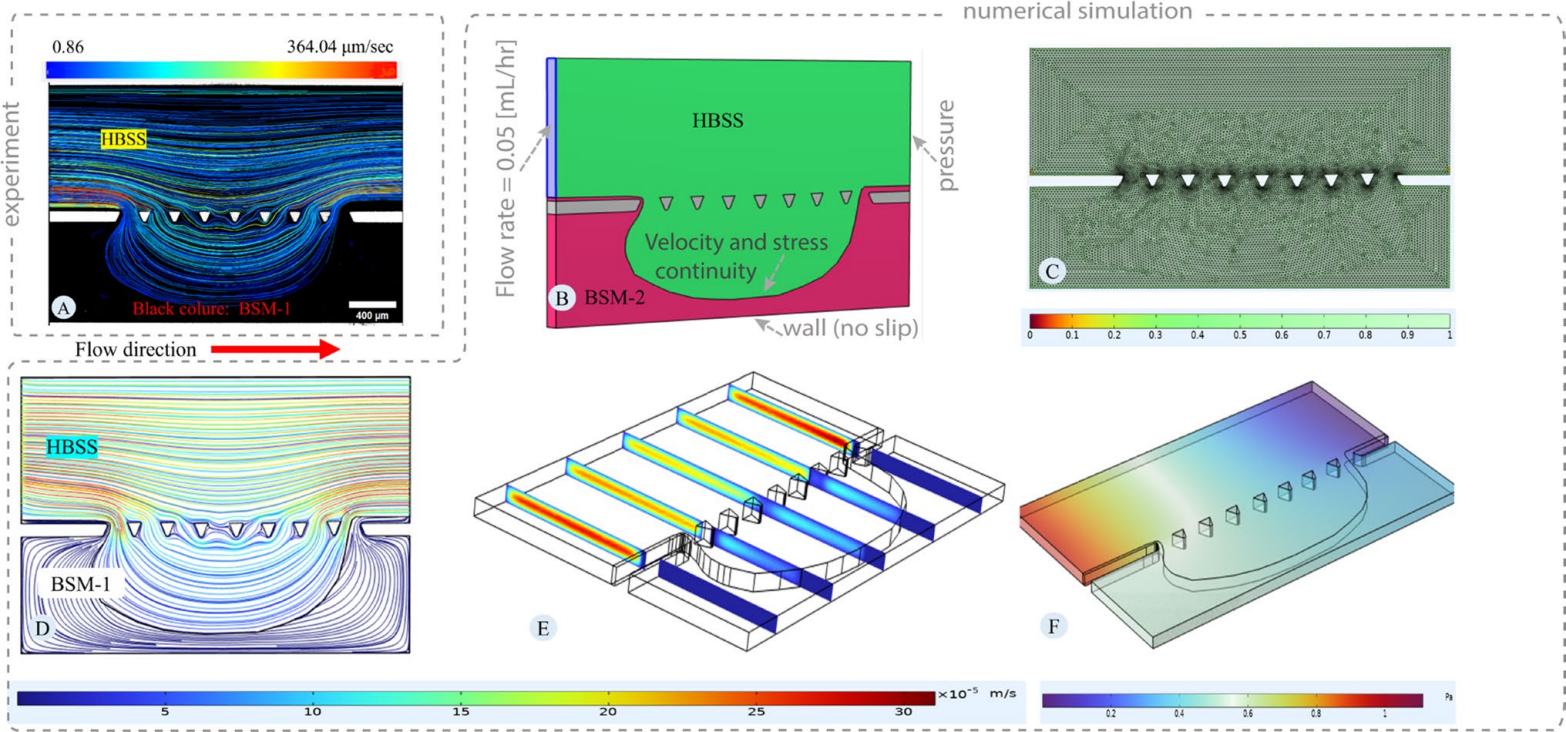
**Velocity field for BSM-1:** Panel **A** shows the velocity distribution considering the tracks of the green and red particles. Tracking is done for the experiment with BSM-1 from 730 to 790 s. Panel **B** presents the 3D geometry utilized for simulation and boundary conditions. Panel **C** shows the mesh quality for the numerical simulation. Panel **D** illustrates the velocity streamlines, indicating the flow pattern and velocity magnitude. Panel **E** depicts the velocity profile over cut planes, showing the variation of velocity in 3D orientation. Panel **F** displays the pressure distribution

Panels A in Fig. 12 depict the average velocity of the tracks observed during the experiment with BSM-1, including particle tracks recorded from 730 to 790 s. Panel A illustrates the trajectory of the green and red particles, delineating the area where HBSS and mucus are flowing and evaluating the velocity profile within this region. Like the previous simulation for BSM-2, the objective here is to mimic the results in Panel A of Fig. 12. Panel B shows the geometry created based on Panel A with boundary conditions. The simulation was conducted using the equations provided in the In silico study section. Both BSM-1 and HBSS were considered incompressible fluids. The physics-based meshing technique of COMSOL was employed, as illustrated in Fig. 12-C. Verification of numerical mesh quality was conducted using COMSOL's average and minimum mesh quality parameters.

Panel E of Fig. 12 illustrates the velocity variation. The established zone from the experiment serves as a case for simulation to confirm the modelling approach. The results of the simulation reveal that the velocity establishes within the HBSS area, while there is no significant velocity observed in the BSM-1 region, despite the apparent viscosity of BSM-1 and HBSS being close to each other (BSM-1 viscosity approximately 50 times greater than HBSS). This absence of significant velocity in BSM-1 can be attributed to the established interface, which can be termed a stable interface, where the shear induced by the flow of HBSS to BSM-1 is not sufficient to cause movement. In the HBSS channel, velocity near the walls diminishes to zero due to the no-slip boundary condition. In Panel D, streamlines in the model are depicted. These streamlines capture velocities both near the walls and in the center of the channel, resulting in non-smooth coloring that represents velocity variations along the streamlines. The streamlines in the numerical simulation depict the flow pattern within the HBSS and BSM-1, which is consistent with the tracking of particles observed in the in-vitro investigation (Panel A). Pressure distribution for maintaining the flow is provided in Panel F.

## Discussion

The rheological properties of mucus play a fundamental role in its hydration, lubricating abilities, and function as a selective barrier to nutrients, medicines, and pathogenic [32, 33, 54]. Mucus is a mesh-like structure [32] and at the macroscopic level, behaves like a non-Newtonian viscoelastic fluid with viscous (flowing) and elastic (deformation resistance) properties [33]. At the microscopic level, mucus has a low viscosity fluid behavior [38, 55, 56]. Several studies investigate the rheology of native mucus and synthetic mucus models to measure viscosity, storage, and loss modulus [34, 57]. There is a wide range of reported data for the rheology of intestinal mucus depending on several factors, including the specific intestinal section [50], pH [57], the type of mucin protein [34], various diseases (human bronchial epithelial mucus and cystic fibrosis mucus [58]), and the abundance of bacteria [32–34]. Because of the variation in the viscosity and elastic properties of the mucus, we used BSM-2 as a representation of the elastic and high viscose mucus models which aligns more closely with that of intestinal mucus [31, 48–50, 58] and BSM-1 as a representation of the low viscosity mucus models which mimics the mucus in the lose region and presence of diseases and bacteria [32–34, 58].

A variety of mucus models, including purified mucin, native mucus, cell culture (mucus-producing cell lines), and biosimilar mucus [29, 59], have been used for barrier studies. The most accurate representation of the intestinal mucus barrier would involve using human intestinal mucus. However, due to the difficulty in obtaining human samples, animal-sourced mucus, particularly from pigs' small intestines [38, 50], is often used as a proxy. Krupa et al. showed that pig intestinal mucus could be a suitable model for human small intestinal mucus studies [38]. However, the use of animal-sourced mucus is limited by ethical considerations and methodological differences. Although the use of biosimilar mucus is ethically preferable to using fresh animal-sourced mucus models, animal-sourced mucus (Mucin Type III), typically extracted from pig gastric mucus, is still required to create BSM [31]. To minimize reliance on animal-sourced mucus models, biopolymer-based mucus models could be used [60]. However, the validity of these biopolymer-based models must be rigorously benchmarked against native tissues.

The small intestine epithelium continually strives to achieve a balance between efficient nutritional absorption and minimal damage from ingested, secreted, and resident agents [61, 62]. A vital part of intestinal epithelial protection is the secretion of mucins by specialized cells known as goblet cells (GCs), which form a gel to uphold surface hydration and alleviate mechanical stress. This mucus layer serves as a diffusion barrier, proving pH and antimicrobial gradients that safeguard underlying cell surfaces [63, 64]. To sustain its protective role, the mucus undergoes constant renewal through secretion [65–67]. As showed the renewal of the mucus layer can vary from several minutes to hours [68, 69]. Studies suggest that mucus spontaneously grows at a rate of around 240 μm/h in humans and 100 μm/h in mice [63, 67, 70]. During this study, the flow rate is maintained at 0.05 ml/hr, leading to comparable velocities in the intestine during large contractions [40], which leads to the dislodgement rate of 0.66 ± 0.088 μm/sec. It is noted that the renewal rate in the intestine is not as rapid as observed in the microfluidic model. Dislodgement of mucus in the intestine facilitated by the induced shear from the flowing lumen material, pH variations between the lumen material and the mucus, and bacterial activity within the lumen. Shear-induced dislodgement is facilitated by peristaltic motion and contractions of the intestinal walls, involving both forward and backward flows [71–74], leading to mitigating the duration and order of the induced shear stress. However, conducting experiments of this nature poses challenges. Therefore, this study opted for a simplified flow condition to offer validation for numerical simulations. A validated numerical approach holds promise as a tool for analysing bodily conditions.

The phenomenon akin to mucus dislodgement in the intestine finds its counterpart in the respiratory tract, known as mucociliary clearance. Extensive studies have delved into this phenomenon through numerical simulations [75–79]. Comparing the outcomes of in-silico simulations with those of microfluidic devices underscores the capability of numerical simulations to replicate on-chip conditions by solving mathematical models. The gastrointestinal tract's mechanically dynamic environment, involving flow and mixing of liquid, solid, and semi-solid digest, along with diverse forms of contractile activity, results in a large variation in shear forces within the small intestine in terms of their magnitude and their duration [71–74, 80–83]. These forces contribute to mucus removal due to induced shear stress. Investigations into mucus removal must incorporate physiologically relevant shear patterns, chyme viscosity and density. One possible approach to studying mucus removal is to integrate the proposed numerical method with existing research aimed at simulating intestinal wall movement, as evidenced by studies from [72–74, 81–84]. By considering these studies as a foundation for understanding chyme movement, the proposed numerical method in this study can calculate induced shear stress on the mucus layer, supplying a platform for measuring mucus removal under physiologically relevant conditions. This could involve employing moving mesh or two-phase flow modeling techniques to simulate the intricate dynamics of the gastrointestinal environment.

Our microfluidic platform offers a new approach to studying the intestinal mucus barrier properties under controlled conditions. This facilities real time monitoring of mucus interactions with luminal materials, providing valuable insights into the penetration of drugs, particles and pathogens. By enabling the use of different mucus viscosities and compositions, the platform supports investigations into pathological conditions, bacterial interactions, and disease-specific mucus dynamics. Such capabilities are critical for developing targeted drug delivery systems and advancing personalized medicine.

Numerical modelling also holds promise for scaling these insights to in silico studies of drug absorption studies. In-silico methods are favoured for their cost-effectiveness and reproducibility [85]. The examination of drug uptake in the intestine through in-silico methods is a complex, multiscale problem that requires consideration of various mechanisms, including solving an existing digestion model for drug availability and concentration in the small intestine [86, 87] and addressing the hydrodynamics of flow on the lumen side of the intestine with models that account for contraction, peristalsis, and the effect of villi [88–91]. At this stage, the proposed modeling approach with viscosity models is applicable for modeling fluid flow within the mucus layer. Our model needs to be coupled with a viscosity-dependent mass transfer equation, offering a robust foundation for predicting drug permeability through a mucous barrier [19]. The diffusion coefficient of substances is a crucial factor for solving the mass transfer equation, as it determines the rate at which substances diffuse into the mucus layer. Through sensitivity analysis, we demonstrated how variations in the diffusion coefficient can significantly impact the barrier properties of the mucus layer [19].

## Opportunities and future directions

Although the proposed microfluidic approach to mimic the intestinal mucus barrier takes us a step closer to replicating physiological conditions, there are still several aspects that need to be addressed before these systems can be considered reliable in vitro predictive models. Future efforts should focus on addressing the following limitations:

- Integration of the mucus-producing cells: Goblet cells, which are responsible for secreting mucus, are essential for maintaining mucus homeostasis. Incorporating these cells into microfluidic systems would enable continuous mucus replenishment, compensating for mucus dilution and dislodgement along with pathogens, thus better mimicking physiological conditions.
- Incorporation of intestinal topology into microfluidic models: the complex structure of the intestinal villi influences flow streamlines and mucus layer thickness. Incorporating villi-like features into microfluidic systems could significantly enhance their precision and physiological relevance
- Simulating the back-and-forth flow regime of the intestine: although the net flow in the intestine is forward, the wall contraction of intestinal generate a back-and-forth motion, reducing shear stress on the mucus layer and alter its resistance to lumen flow. Introducing this dynamic flow behavior into microfluidic systems would better mimic physiological conditions.
- Incorporating realistic intestinal geometries in numerical simulations: Verified numerical simulations should reflect the true complexity of the intestine, including villi structures, cylindrical shapes, regional diameter variations, and the total length of the intestine, to capture the intricacies of the intestinal environment accurately.
- Developing a comprehensive viscosity model: a generalized viscosity model is needed to account for variations in mucus viscosities based on its location (e.g., jejunum, ileum, etc.), proximity to the epithelial layer, and the changes in viscosity due to diseases. Such model is essential for robust conducting comprehensive numerical simulations of intestinal processes.

## Conclusion

In conclusion, this study introduces a novel microfluidic platform for investigating the interplay between intestinal mucus and luminal material, emphasizing the crucial role of shear stress in mucus dislodgement and penetration. By integrating biosimilar mucus models with validated numerical simulations, the research highlights the impact of mucus viscosity and viscoelasticity on barrier properties and demonstrates the effectiveness of shear-thinning constitutive models in predicting mucus behavior. This platform serves as a versatile tool for real-time studies of drug transport, pathogen interactions, and mucus dislodgement, bridging the gap between in vitro and in vivo research. These advancements lay the foundation for improved in silico modeling of drug absorption and the development of personalized therapies, enhancing our ability to replicate physiological conditions in the laboratory.

## Data Availability

The datasets generated during and/or analysed during the current study are available from the corresponding author on reasonable request.
